# Special Issue: *Candida* and Candidiasis

**DOI:** 10.3390/jof4030074

**Published:** 2018-06-21

**Authors:** Jeniel E. Nett

**Affiliations:** Departments of Medicine and Medical Microbiology & Immunology, University of Wisconsin, Madison, WI 53706, USA; jenett@medicine.wisc.edu

**Keywords:** *Candida*, candidiasis, virulence, biofilm, diagnosis, hyphae, phospholipid

## Abstract

This special issue highlights emerging topics related to *Candida*, the most prevalent fungal pathogen in the hospital setting. The advantages and limitations of new, non-culture based diagnostic techniques are discussed. The issue reviews mammalian and non-mammalian infection models. The manuscripts present updates on several molecular mechanisms of pathogenicity, including filamentation, biofilm formation, and phospholipid production.

*Candida*, the most common nosocomial fungal pathogen, causes a diverse spectrum of diseases. It frequently exists as a commensal fungus colonizing the gastrointestinal tract and can cause mucosal disease, such as oroesophageal or vaginal candidiasis. With immunosuppression, *Candida* becomes an invasive pathogen, leading to disseminated disease with mortality approaching 40% [1,2]. As culture-based diagnostic tests for invasive candidiasis lack sensitivity, diagnosis may be delayed, contributing to increased morbidity and mortality [3]. The occurrence of drug-resistant strains poses an additional obstacle to treatment [4]. Furthermore, we are seeing the emergence of *Candida auris*, which is causing antifungal-resistant outbreaks worldwide [5,6]. *Candida* spp. possess a multitude of virulence factors, which permit attachment, facilitate tissue invasion, and promote the formation of resilient biofilm communities on medical devices and host surfaces. Further understanding of *Candida* pathogenicity will aid in the development of improved diagnostic and therapeutic approaches. 

While blood cultures remain the standard method for the diagnosis of invasive candidiasis, it is estimated that 50% of cases may be missed by use of this technique alone [3]. In this issue, Clancy and Nguyen discuss the role of non-culture based techniques for the diagnosis of candidiasis, including assays to detect mannan, anti-mannan antibodies, *Candida albicans* germ tube antibodies, and 1,3-β-d-glucan, as well as the T2Candida nanodiagnostic panel and polymerase chain reactions (PCR) [7]. These non-culture based methods offer improved sensitivity and the promise of more rapid diagnosis. Despite the high performance of many of these tests, cultures remain necessary for obtaining antifungal susceptibility data. The possible unintended consequences of using non-culture diagnostics are also examined, including the potential for inappropriate antifungal use if these tests are ordered for patients with a low pre-test probability of invasive candidiasis. 

Animal models have been critical for the advancement of our understanding of *Candida* pathogenicity. A manuscript by Segal and Frenkel highlights the use of both mammalian and non-mammalian models for studying pathogenesis [8]. Mammalian models most closely mimic clinical candidiasis, with murine models the most frequently utilized to model both systemic and mucosal disease. Various methods of replicating host immunocompromise, such as neutropenia, can be employed. The authors discuss diverse uses for the models, including the study of host immunity, virulence traits, and antifungal pharmacokinetics. While non-mammalian models may be less representative of candidiasis in humans, these systems are often less costly and ideal for high throughput studies. Candidiasis models in the fruit fly *Drosophila melanogaster*, the larvae of the moth *Galleria mellonella*, and the free-living nematode *Caenorhabditis elegans* are described and compared to mammalian models.

The ability of *Candida* spp. to form biofilm communities on medical devices and biotic surfaces is an increasingly recognized virulence trait. In this issue, Kean and colleagues discuss biofilm formation by *Candida*, focusing on the current models of study. They review the molecular aspects underpinning the transition to the biofilm lifestyle and the acquisition of antifungal drug tolerance [9]. The authors highlight the inherent heterogeneity associated with these communities, which contain diverse microenvironments and form in distinct anatomic niches. Furthermore, the capacity to form biofilms varies greatly among *Candida* spp., as well as among clinical isolates of the same species. The importance of including a variety of clinical isolates for biofilm studies is also discussed. 

Two manuscripts in this issue provide updates on our understanding of hyphal morphogenesis. Components of this pathway are potential drug targets, as filamentation is unique to fungi and critical for the pathogenesis of *C. albicans*. The review by Desai divides the formation of hyphae into 3 steps, which include hyphal initiation, elongation, and directionality maintenance [10]. The mechanistic regulation of each step is discussed, as are the environmental cues triggering filamentation. The concepts of both fungal-mediated tissue invasion and host-assisted uptake are reviewed. Lin and Chen also provide an update on the signaling pathways governing morphogenesis in *Candidia* [11]. Their review focuses on the cyclic adenosine monophosphate/protein kinase A (cAMP/PKA) cascade, which regulates hyphal growth as well as white–opaque switching in *C. albicans*. Similarities and differences in these regulatory pathways between *C. albicans* and its close relative *C. tropicalis* are highlighted. Identifying signaling components conserved among *Candida* spp., and even among fungi, is essential for recognizing potential broad-spectrum drug targets. 

This issue also features a review of phospholipid biosynthesis in *C. albicans*, focusing on two aminophospholipids, phosphatidylserine and phosphatidylethanolamine, and their role in microbial pathogenesis [12]. Cassilly and Reynolds describe the current knowledge of the phosphatidylserine and phosphatidylethanolamine synthesis pathways in *C. albicans* and provide a comparison to these biosynthetic pathways in other eukaryotes, as well as bacteria. The potential for targeting aminophospholipid pathways for drug development is discussed. In particular, the phosphatidylserine synthase of *C. albicans* appears to be conserved among fungi and is structurally distinct from mammalian phospholipid synthases. 

I would like to take the opportunity to thank each of the authors for contributing their expertise to this Special Issue on *Candida* and Candidiasis.
